# Proinflammatory Cytokine Preconditioning Enhances the Therapeutic Potency of Different Types of MSCs in Inflammation

**DOI:** 10.3390/ijms27094090

**Published:** 2026-05-02

**Authors:** Lanzhi Liu, Juan Fandiño, Abigail J. M. Warren, Rui Shi, Ignacio Sallent, Shanshan Du, Sean D. McCarthy, Claire Masterson, Matt Angel, Christopher B. Rohde, John G. Laffey, Daniel O’Toole

**Affiliations:** 1 CÙRAM, SFI Research Centre for Medical Devices, University of Galway, H91 W2TY Galway, Ireland; l.liu8@universityofgalway.ie (L.L.); sean.mccarthy@universityofgalway.ie (S.D.M.); jlaffey@universityofgalway.ie (J.G.L.); daniel.otoole@universityofgalway.ie (D.O.); 2 Discipline of Physiology, School of Pharmacy and Medical Sciences, University of Galway, H91 TK33 Galway, Ireland; r.shi5@universityofgalway.ie (R.S.); isallent@ub.edu (I.S.); shanshan.du@universityofgalway.ie (S.D.); claire.masterson@universityofgalway.ie (C.M.); 3 Discipline of Anaesthesia, School of Medicine, University of Galway, H91 CF50 Galway, Ireland; a.warren4@universityofgalway.ie; 4 Immunology Unit, Department of Pathology and Experimental Therapies, Faculty of Medicine and Health Sciences–Bellvitge Biomedical Research Institute (IDIBELL), University of Barcelona, 08908 Barcelona, Spain; 5 Factor Bioscience Inc., Cambridge, MA 02141, USA; matt.angel@factorbio.com (M.A.); chris.rohde@factorbio.com (C.B.R.)

**Keywords:** BM-MSC, iMSC WT, iMSC B2M KO, inflammation

## Abstract

Mesenchymal stromal cells (MSCs) have shown immunomodulatory effects and great promise in many inflammatory diseases such as acute respiratory distress syndrome (ARDS). However, several barriers to translation remain such as cell availability and potency. This study evaluates the therapeutic potentials of three types of MSCs, bone marrow-derived MSCs (BM-MSC), the human induced pluripotent stem cell-derived MSC wild type (iMSC WT) and β2 microglobulin-knockout iMSCs (iMSC B2M KO) with or without proinflammatory cytokine preconditioning. BM-MSC, iMSC WT and iMSC B2M KO were preconditioned with a proinflammatory cytokine cocktail (Cytomix: IL-1β, IFN-γ and TNF-α). Immunoregulatory biomarkers were analysed by flow cytometry and cytokines released by ELISA. MSC antimicrobial properties were analysed via CFU assays while the MSCs’ immunomodulatory effects were evaluated using macrophage activation and T cell proliferation assays. Proinflammatory cytokine preconditioning enhanced the therapeutic potency of all three types of MSCs by increasing immunomodulatory marker expression, enhancing the antimicrobial effects and improving MSC-mediated inhibition of T cell proliferation. These findings provided new insights into the therapeutic potencies of MSCs in inflammation. Further studies are required for in vitro characterisation of the MSCs and in vivo efficacy verification of these MSCs prior to their clinical application.

## 1. Introduction

Inflammation is a protective immune response to infection or injury, which aims to kill pathogens and aid tissue repair [1,2]. However, dysregulated immune responses, particularly of the innate and adaptive immune system, can lead to various inflammatory or autoimmune diseases such as sepsis, acute respiratory distress syndrome (ARDS), asthma, inflammatory bowel diseases (IBDs), and graft-versus-host disease (GvHD) and can cause acute or chronic tissue damage [1,3,4,5]. Anti-inflammatory therapies including corticosteroids, immunosuppressants and monoclonal antibodies are commonly applied to treat these diseases, but there are limitations in their application such as drug resistance and adverse effects [6,7,8]. New therapeutic options are required to modulate immune responses in inflammatory diseases, and mesenchymal stromal cells (MSCs) have emerged as promising alternatives due to their anti-inflammatory, immunomodulatory, antimicrobial and regenerative properties [9,10].

MSCs were first isolated from bone marrow and described by Friendenstein et al. as hematopoietic cells that could differentiate to bone [11]. In 2006, the International Society for Cellular Therapy (ISCT) proposed a minimum criteria to identify MSCs including: (1) their adherence to plastic in standard culture conditions; (2) presence of the markers CD105, CD73 and CD90 and absence of the markers CD45, CD34, CD14/CD11b, CD79α/CD19 and HLA-DR; and (3) in vitro differentiation into osteoblasts, adipocytes and chondroblasts [12]. Previous research has shown that MSCs regulate both innate and adaptive immune responses via their secretome and crosstalk with immune cells including macrophages, natural killer cells, neutrophils and lymphocytes [1,10]. Bone marrow-derived MSCs (BM-MSCs) are one of the most widely described types of MSCs and have shown therapeutic effects in different inflammatory diseases including GvHD, ARDS, sepsis and IBD [13,14,15]. Donor variability is a critical barrier for the application of BM-MSCs in pre-clinical and clinical studies, which necessitates BM-MSC characterisation using in vitro potency assays [16,17]. Moreover, to overcome the limitations of BM-MSCs such as cell population heterogeneity, cell senescence, and loss of multipotency over continuous passages, new sources of MSCs, human induced pluripotent stem cell-derived MSCs (iMSCs), are being investigated [18]. As a potential infinite and more homogenous cell source, these cells have been previously demonstrated to alleviate epithelial cell dysfunction in an asthma model [19] and to modulate immune response by inhibiting the proliferation and activation of T cells in a systemic inflammatory model [20]. To reduce the immunogenicity of iMSCs and improve the therapeutic potential of iMSCs, a β2 microglobulin knockout iMSC cell line (iMSC B2M KO) that does not express human leukocyte antigen class I complex (HLA-I), has been developed, while maintaining the phenotype, multipotency and immunomodulatory characteristics of iMSCs [21].

A growing body of evidence suggests that the host environment, especially the active inflammatory environment, plays a vital role in the initiation of MSC immunoregulation and the efficacy of MSC-based therapies [22,23]. Previous studies show that inflammatory cytokine preconditioning can alter the phenotype and functions of MSCs: IL-1β improved MSC immune regulation and targeted migration abilities, interferon (IFN)-γ enhanced MSC immunomodulatory capability, and tumour necrosis factor (TNF)-α promoted MSC chemotaxic potential, while anti-inflammatory cytokines such as IL-4 and IL-10 had little to no effects on MSC immune-related functions [24,25,26]. The combination of different inflammatory cytokines showed various effects on different types of MSCs. IFN-γ and TNF-α upregulated the expression of TNF-α-stimulated gene-6 (TSG-6) in umbilical cord-derived MSCs (UC-MSCs) to reduce neutrophil infiltration [27]. TNF-α and IL-1β enhanced BM-MSC’s capability of suppressing the proliferation of lymphocytes in a nitric oxide-dependent manner [28], while the combination of IL-1β, IFN-γ and TNF-α increased the inhibitory effect of UC-MSCs on NF-κB activation and reduced donor-dependent heterogeneity and improved MSC immunomodulatory properties [29,30]. These studies indicate that cytokines, as a part of the disease microenvironment, can alter MSC phenotype and function and imply that using functional assays to assess MSC therapeutic potencies prior to in vivo studies is critical to MSC donor selection. In this study, we sought to apply a series of assays to evaluate the effects of a cocktail of proinflammatory cytokines (IL-1β, IFN-γ and TNF-α) on the physiology and therapeutic potencies of BM-MSC, iMSC WT and iMSC B2M KO.

## 2. Results

### 2.1. Cytomix Modulates the Immunophenotype of BM-MSC, iMSC WT and iMSC B2M KO

**Figure 1:**
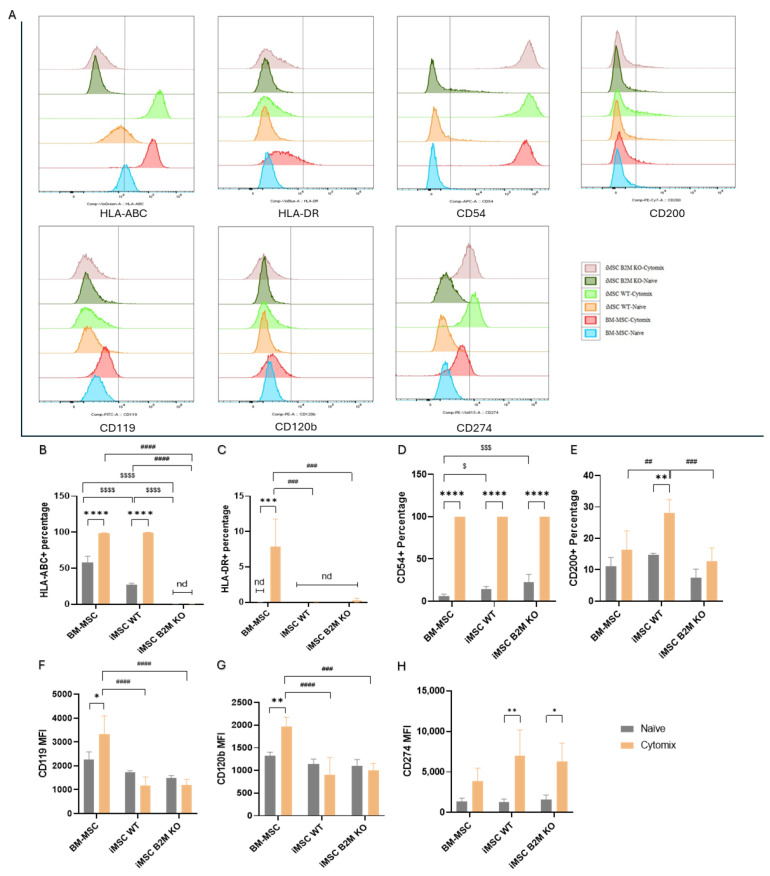
Cytomix increased the expression of immunomodulatory markers on BM-MSCs, iMSCs WT and iMSCs B2M KO. The gating strategy is shown in (Supplementary Figure S2). (**A**) Histogram charts of the expression of the immunomodulatory markers on BM-MSCs, iMSCs WT and iMSCs B2M KO. (**B**–**H**) Cytomix significantly increased the expression levels of HLA-ABC on BM-MSCs and iMSCs WT, HLA-DR on BM-MSCs, CD54 on all types of MSCs, CD200 on iMSCs WT, CD119 and CD120b on BM-MSCs, and CD274 on iMSCs WT and iMSCs B2M KO. Representative results of three independent experiments are shown. Results are shown as mean ± SD (*n* = 3 in each group) and analysed by two-way ANOVA with Šídák’s multiple comparisons test or Tukey’s multiple comparisons test. */**/***/**** represent comparisons of naïve vs. Cytomix in each type of MSC; $/$$$/$$$$ represent comparisons of BM-MSCs vs. iMSCs WT vs. iMSCs B2M KO in the naïve group; ##/###/#### represent comparisons of BM-MSCs vs. iMSCs WT vs. iMSCs B2M KO in the Cytomix group with significance levels of *p* < 0.05/0.01/0.001/0.0001, respectively. Nd represents not detectable.

BM-MSC, iMSC WT and iMSC B2M KO tested positive (≥95%) for MSC-positive markers CD73, CD90 and CD105 and negative (≤2%) for MSC-negative markers CD45, CD34, CD11b, CD19 and HLA-DR (Supplementary Figure S1). Cytomix significantly increased the expression level of HLA-ABC, the primary type of class I human leukocyte antigens, on BM-MSCs and iMSCs WT (Figure 1B). No HLA-ABC was detected on either naïve or Cytomix-stimulated iMSCs B2M KO (Figure 1B). Among the naïve MSCs, BM-MSCs had a significantly higher expression level of HLA-ABC than iMSCs WT and iMSCs B2M KO (Figure 1B). Cytomix significantly increased the expression level of HLA-DR, a subtype of class II human leukocyte antigen, on BM-MSCs but not on iMSCs WT or iMSCs B2M KO (Figure 1C). CD54 was detected at a significantly higher level on all Cytomix-activated MSCs compared to naïve MSCs (Figure 1D). In naïve cells, the level of CD54 was significantly higher on iMSCs compared to BM-MSCs (Figure 1D). Compared to naïve MSCs, respectively, Cytomix significantly increased the levels of CD200 on iMSCs WT but not on the other two types (Figure 1E). Cytomix significantly increased the median fluorescence intensity (MFI) of CD119 and CD120b on BM-MSCs compared to iMSCs WT and iMSCs B2M KO (Figure 1F,G). However, Cytomix significantly increased the MFI of CD274 (Figure 1H) on iMSCs WT and iMSCs B2 M KO relative to the BM-MSC group.

### 2.2. Cytomix Alters the Secretome from BM-MSCs, iMSCs WT and iMSCs B2M KO

**Figure 2:**
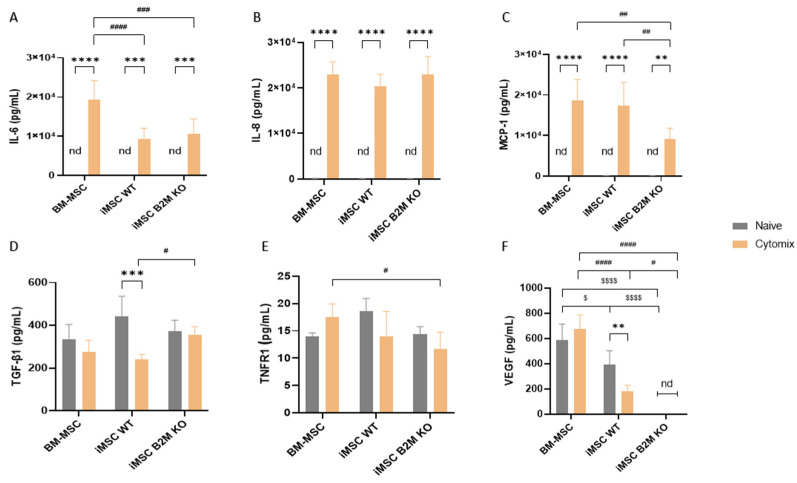
Cytomix changed the secretome of BM-MSC, iMSC WT and iMSC B2M KO. (**A**–**F**) Cytomix significantly increased the levels of IL-8, IL-6 and MCP-1 in the CM from BM-MSC, iMSC WT and iMSC B2M KO and significantly decreased the levels of TNFR1, TGF-β1 and VEGF in the CM from iMSC WT. Representative results of four independent experiments are shown. Bars represent mean ± SD (*n* = 4 in each group) and analysed by two-way ANOVA with Šídák’s multiple comparisons test to compare naïve vs. Cytomix in each type of MSC or Tukey’s multiple comparisons test to compare BM-MSC vs. iMSC WT vs. iMSC B2M KO in naïve or Cytomix group. **/***/**** represents comparisons of naïve vs. Cytomix in each type of MSCs; $/$$$$ represents comparison of BM-MSC vs. iMSC WT vs. iMSC B2M KO in the naïve group; #/##/###/#### represents comparison of BM-MSC vs. iMSC WT vs. iMSC B2M KO in the Cytomix group with significance levels of *p* < 0.05/0.01/0.001/0.0001, respectively. nd represents not detectable.

Cytokine analysis of the secretome demonstrated that the proinflammatory cytokines IL-6, IL-8 and MCP-1 were not detectable in the CM from naïve BM-MSCs, iMSCs WT or iMSCs B2M KO (Figure 2A–C). Cytomix preconditioning significantly increased the level of IL-6 in the CM from all of the types of MSCs compared to naïve, but the Cytomix-stimulated BM-MSCs produced a significantly higher level of IL-6 compared to the two types of iMSCs (Figure 2A). Cytomix preconditioning significantly increased the level of IL-8 secreted by BM-MSCs, iMSCs WT and iMSCs B2M KO in comparison to naïve MSCs (Figure 2B). Cytomix significantly increased the level of MCP-1 production from the three types of MSCs, with a significantly higher level in the CM from the BM-MSCs and the iMSCs WT compared to the iMSCs B2M KO (Figure 2C).

Regarding anti-inflammatory cytokines, Cytomix significantly decreased the levels of TGF-β1 and TNFR1 in the CM from iMSCs WT (Figure 2D,E). Among naïve MSCs, the CM from iMSCs WT presented a significantly higher level of TGF-β1 than the other two MSC types. Among Cytomix-stimulated MSCs, the CM from iMSCs WT presented a significantly reduced level of TGF-β1, compared to iMSCs B2M KO (Figure 2D). In naïve MSCs, iMSCs WT secreted a higher level of TNFR1 than BM-MSCs and iMSCs B2M KO; among Cytomix-stimulated MSCs, the CM from BM-MSCs had a higher level of TNFR1 than iMSCs WT and iMSCs B2M KO (Figure 2E). No VEGF was detected in the CM from iMSCs B2M KO (Figure 2F). Cytomix significantly decreased the level of VEGF secreted by iMSCs WT. Naïve and Cytomix-stimulated BM-MSCs secreted a significantly higher level of VEGF compared to iMSCs WT and iMSCs B2M KO (Figure 2F).

### 2.3. Cytomix Enhances the Antibacterial Effects of BM-MSCs, iMSCs WT and iMSCs B2M KO

**Figure 3:**
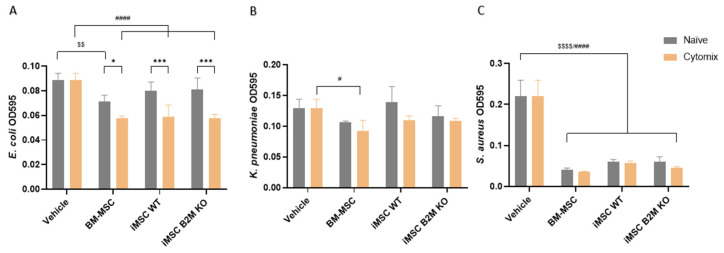
Cytomix enhanced the antimicrobial effects of BM-MSCs, iMSCs WT and iMSCs B2M KO. (**A**,**B**) Cytomix significantly enhanced the inhibitory effects of CM from BM-MSCs, iMSCs WT and iMSCs B2M KO on the proliferation of *E. coli*, and from BM-MSCs on the proliferation of *K. pneumoniae*. (**C**) Both naïve and Cytomix-stimulated CM from the three types of MSCs were demonstrated to reduce the proliferation of *S. aureus*. Representative results of four independent experiments are shown as mean ± SD (*n* = 4 in each group) and analysed by two-way ANOVA with Šídák’s multiple comparisons test to compare naïve vs. Cytomix in each type of MSC or Tukey’s multiple comparisons test to compare BM-MSCs vs. iMSCs WT vs. iMSCs B2M KO in the naïve or Cytomix group. */*** represent comparisons of naïve vs. Cytomix in each type of MSC; $$/$$$$ represent comparisons of BM-MSCs, iMSCs WT and iMSCs B2M KO vs. vehicle in the naïve group; and #/#### represent comparisons of BM-MSCs, iMSCs WT and iMSCs B2M KO vs. vehicle in the Cytomix group with significance levels of *p* value < 0.05/0.01/0.001/0.0001, respectively. Vehicle represents the basal medium used for CM.

Gram-negative *Escherichia coli* (*E. coli*), *Klebsiella pneumoniae* (*K. pneumoniae*) and Gram-positive *Staphylococcus aureus* (*S. aureus*) were used to evaluate the antibacterial effects of the CM from the three MSC types. Naïve BM-MSC CM significantly inhibited *E. coli* growth, whereas naïve iMSC WT and iMSC B2M KO CM showed no significant effect (Figure 3A). Cytomix preconditioning enhanced the antibacterial activity of CM from all three MSC types against *E. coli* (Figure 3A). In contrast, no inhibition of *K. pneumoniae* growth was observed with naïve CM from any MSC type. Cytomix preconditioning selectively enhanced antibacterial activity only in BM-MSC CM (Figure 3B). Regarding the Gram-positive bacteria *S. aureus*, naïve CM from all three MSC types significantly inhibited bacterial growth, with no further improvement observed after cytomix preconditioning (Figure 3C).

### 2.4. Cytomix Reduces the MSCs’ Inhibitory Effects on NF-κB Pathway Activation in Macrophages While Enhancing the MSC Effect on Macrophage Phagocytosis

**Figure 4:**
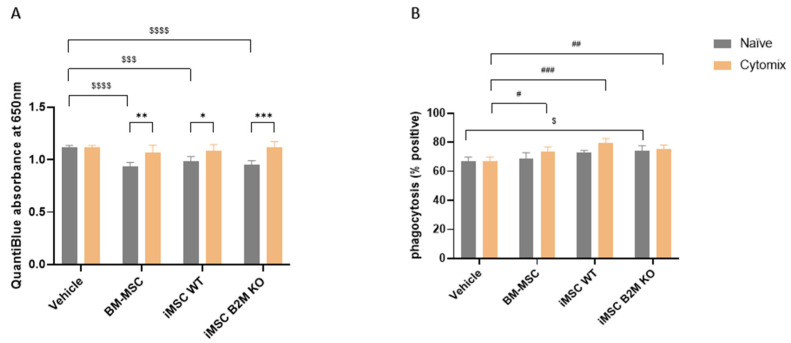
Cytomix alters MSC effects on the NF-κB activation and phagocytosis of macrophages. (**A**) CM from naïve BM-MSCs, iMSCs WT and iMSCs B2M KO significantly inhibited LPS-induced NF-κB activation in THP-1-derived macrophages, while Cytomix reduced the effect of the three types of MSCs. (**B**) The images of phagocytosis are shown in (Supplementary Figure S3). CM from naïve iMSCs B2M KO significantly increased the phagocytosis-positive cells percentage of THP-1-derived macrophages, while CM from Cytomix-stimulated BM-MSCs, iMSCs WT and iMSCs B2M KO all significantly increased the phagocytosis-positive percentage of these macrophages. Representative results of three independent experiments are shown. Results are shown as mean ± SD (*n* = 3 in each group). QuantiBlue was analysed by two-way ANOVA with Dunnett’s multiple comparisons test to compare CM from all naïve and Cytomix-stimulated MSCs vs. vehicle; */**/*** represent a *p* value < 0.05/0.01/0.001. Phagocytosis was analysed by two-way ANOVA with Šídák’s multiple comparisons test to compare naïve vs. Cytomix in each type of MSC or Dunnett’s multiple comparisons test to compare BM-MSCs, iMSCs WT and iMSCs B2M KO vs. vehicle in the naïve or Cytomix group. $/$$$/$$$$ represents comparisons of BM-MSC, iMSC WT and iMSC B2M KO vs. vehicle in the naïve group with a significance of *p* value < 0.05/0.001/0.0001; #/##/### represent comparisons of BM-MSCs, iMSCs WT and iMSCs B2M KO vs. vehicle in the Cytomix group with significance levels of *p* value < 0.05/0.01/0.001. Vehicle was the basal media used to make CM.

CM from naïve BM-MSCs, iMSCs WT and iMSCs B2M KO significantly decreased the activation level of the NF-κB pathway, as measured using THP-1 reporter cells in which absorbance at 650 nm reflects NF-κB-dependent SEAP activity, while Cytomix reduced the inhibitory effects of the three types of MSCs on NF-κB pathway activation (Figure 4A). However, Cytomix-stimulated CM from BM-MSCs, iMSCs WT, and iMSCs B2M KO significantly increased the percentage of phagocytosis-positive THP-1-derived macrophages (Figure 4B). Among naïve MSCs, only the CM from iMSCs B2M KO significantly increased the phagocytosis in these macrophages, while BM-MSCs and iMSCs WT demonstrated a trend to increase the phagocytosis, but the effect was not significant (Figure 4B).

### 2.5. Cytomix Enhances the MSCs’ Inhibitory Effects on T Cell Proliferation via CM; Co-Culturing PBMCs with the Naïve or Cytomix-Stimulated MSCs Suppresses T Cell Proliferation in a Dose-Dependent Manner

**Figure 5:**
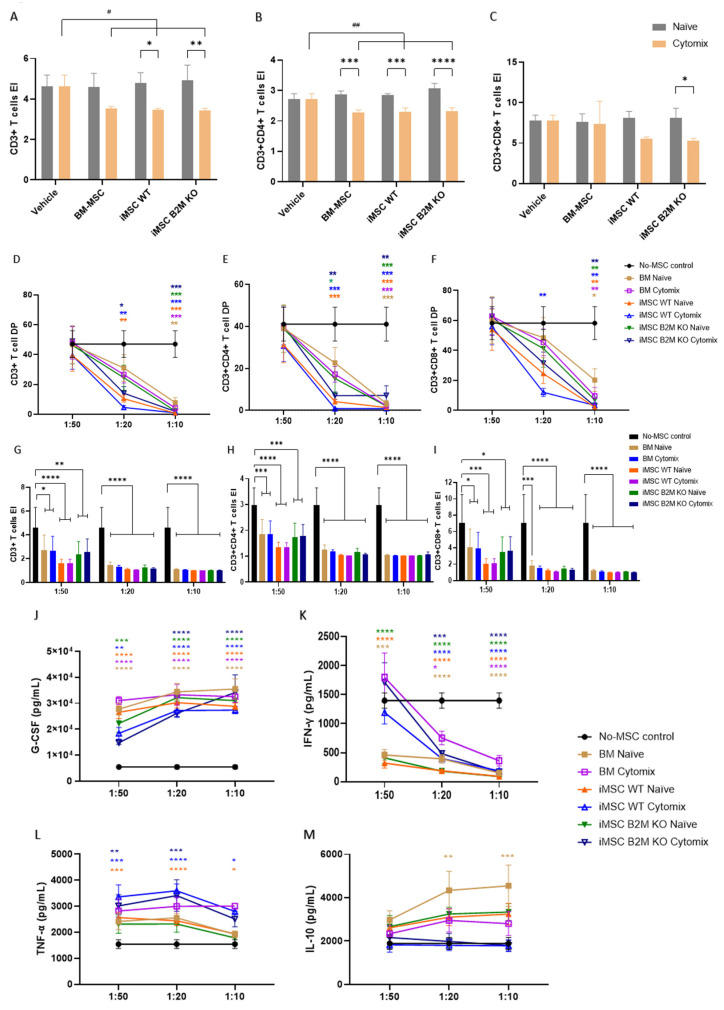
The effects of naïve and Cytomix-stimulated MSCs on T cell proliferation. The gating strategy is shown in Supplementary Figure S4. (**A**–**C**) BM-MSCs, iMSCs WT and iMSCs B2M KO inhibition on the expansion of CD3+, CD3+CD4+ and CD3+CD8+ T cells via CM. Cytomix enhanced the three types of MSCs’ inhibition on the EI of T cells. (**D**–**F**) All types of MSCs significantly inhibited the division of CD3+, CD3+CD4+ and CD3+CD8+ T cells when co-cultured with PBMCs, in a dose-dependent manner. (**G**–**I**) All of the MSCs, naïve or Cytomix stimulated, significantly reduced the EI of CD3+, CD3+CD4+ or CD3+CD8+ T cells, at the lowest ratio of MSCs, with iMSCs WT both naïve and Cytomix-stimulated presenting the strongest inhibition. (**J**) Co-culturing all types of MSCs, naïve or Cytomix-stimulated, increased the production of G-CSF in the media, with a dose-dependent effect. (**K**) All types of MSCs significantly decreased the level of IFN-γ at the ratios of 1:20 and 1:10, while the three types of naïve MSCs significantly decreased it at 1:50. (**L**) Naïve and Cytomix-stimulated iMSC WTs significantly increased the level of TNF-α at the ratios of 1:50, 1:20 and 1:10, while Cytomix-stimulated iMSC B2M KO significantly increased it at the ratio of 1:50 and 1:20. (**M**) Naïve BM-MSCs significantly increased the level of IL-10 production at the ratios of 1:20 and 1:10. Representative results of four independent experiments are shown. Results are shown as mean ± SD (*n* = 3 in CM; *n* = 4 in co-culture, each group), and analysed by two-way ANOVA with Šídák’s multiple comparisons test to compare naïve vs. Cytomix in each type of MSCs (no significance in all of the settings), or Tukey’s multiple comparisons test to compare different ratios of MSCs to PBMCs in each MSC group, or Dunnett’s multiple comparisons test to compare different MSC groups vs. vehicle in the setting of the same ratio. */**/***/**** represent comparisons of each type of MSC vs. vehicle; #/## represent comparisons of different ratios of each cell type with different levels of *p* < 0.05/0.01. EI: expansion index; DP: division percentage.

The CM from the three types of naïve MSCs did not significantly reduce the expansion index (EI) of CD3+, CD3+CD4+ and CD3+CD8+ T cells (Figure 5A–C). The CM from the Cytomix-stimulated MSCs, when compared with the control group, significantly decreased the EI of CD3+ and CD3+CD4+ T cells, but did not significantly decrease CD3+CD8+ T cells (Figure 5A–C). Within each MSC type, Cytomix-stimulated CM reduced the EI compared to the corresponding naïve CM, with significant reduction observed in CD3+CD4+ T cells (Figure 5B), and more selective effects in CD3+ (Figure 5A) and CD3+CD8+ T cells (Figure 5C), particularly for iMSCs WT and iMSCs B2M KO.

When in co-culture conditions, MSC-mediated inhibition of T cell proliferation was strongly dependent on the MSC:PBMC ratio. At a low ratio (1:50), limited effects on division percentage (DP) were observed across all MSC types (Figure 5D–F), whereas increasing MSC numbers (1:20 and 1:10) resulted in a marked, dose-dependent reduction in DP for CD3+, CD3+CD4+, and CD3+CD8+ T cells (Figure 5D–F). At the highest ratio (1:10), all MSC types, regardless of naïve or Cytomix preconditioning, induced a near-complete suppression of T cell division (Figure 5D–F). Similarly, the EI was significantly reduced across all MSC types and ratios (Figure 5G–I), with stronger effects observed at higher MSC doses. Notably, differences between naïve and Cytomix-stimulated MSCs became less apparent in co-culture conditions, particularly at higher ratios, suggesting a convergence of immunosuppressive capacity. At lower ratios, iMSCs WT exhibited a comparatively stronger inhibitory effect on T cell expansion in the three T cell populations. Together, these results indicate that MSC-mediated suppression of T cell proliferation in co-culture is primarily driven by MSC dose, with evidence of a functional saturation effect at higher MSC:PBMC ratios.

The conditioned media from the MSC:PBMC co-culture were used to test G-CSF, TNF-α, IFN-γ and IL-10 (Figure 5J–M) concentration by ELISA. Co-culturing all types MSCs, naïve or Cytomix-stimulated, increased the production of G-CSF in the media, in a dose-dependent manner (Figure 5J). Naïve and Cytomix-stimulated iMSCs WT significantly increased the level of TNF-α at the three different ratios, while Cytomix-stimulated iMSCs B2M KO significantly increased it at the ratios of 1:50 and 1:20 (Figure 5K). All of the MSC types, naïve or Cytomix-stimulated, significantly decreased the level of IFN-γ at ratios of 1:20 and 1:10, while naïve BM-MSCs, iMSCs WT and iMSCs B2M KO significantly decreased IFN-γ at the ratio of 1:50 (Figure 5L). Only naïve BM-MSCs significantly increased the level of IL-10 in the media at the ratios of 1:20 and 1:10 (Figure 5M).

## 3. Discussion

Previous studies have demonstrated that the immunoregulatory mechanisms of MSCs in inflammatory diseases relate to their modulation of innate and adaptive immune cells including macrophages, NK cells and T cells [10]. The local inflammatory microenvironment can alter MSCs phenotypes and functions, making it important to identify both host microenvironment and MSC therapeutic potencies prior to MSC application [31,32]. To improve the efficacy of MSC-based therapies, strategies to address MSC heterogeneity in their immunomodulatory profiles are required, such as preconditioning with inflammatory factors and genetic manipulation [33,34]. Here, a cocktail of proinflammatory cytokines including IL-1β, IFN-γ and TNF-α (Cytomix) and a series of in vitro assays were used to compare the therapeutic potential of a primary MSC and two types of iPS-derived MSCs.

MSC phenotype plays a critical role in their immunomodulatory properties and donor, age and gender are significant influencing factors [35]. In this study, the results show that proinflammatory cytokines altered the phenotypes of BM-MSCs, iMSCs WT and iMSCs B2M KO regarding their immunogenicity, adhesion and migration, and immunomodulatory effects. HLA-A, B and C, as the primary types of class I major histocompatibility complex (MHC) antigen in humans, play important roles in recognising and eliminating self or non-self cells. A previous study showed that allogeneic MSCs can induce the production of CD8+ effector memory T and central memory T cells through HLA-ABC expression [36]. In this study, proinflammatory cytokines significantly increased the expression level of HLA-ABC on BM-MSCs and iMSCs WT which elevated the immunogenicity of these MSCs, while iMSCs B2M KO remained negative for HLA-ABC after Cytomix stimulation. HLA-DR belongs to MHC class II antigens, which trigger T cell activation and adaptive immune response. In published ISCT standards, HLA-DR is a negative marker of MSCs. However, a recent study found that the expression level of HLA-DR did not affect MSC immunomodulatory effects on PBMCs proliferation, making it an obsolete marker for MSC in clinical application [37]. The data show that the proinflammatory cytokines increased the expression of HLA-DR on BM-MSC but not on iMSCs WT or iMSCs B2M KO, although, the positive percentage of HLA-DR in BM-MSC was below 10%. In addition, CD54 (intercellular adhesion molecule 1) plays an important role in leukocyte–endothelial binding and leukocyte transmigration and can improve MSC immunomodulatory capacity by mediating the interaction of MSCs and macrophages [38].

Cytomix significantly increased the level of surface CD54 on three types of MSCs, which indicates the proinflammatory cytokines may facilitate MSC homing and enhance MSCs’ immunomodulatory effects. CD200, a surface immunoregulatory protein, also mediates MSC–macrophage contact and enhances MSC immunosuppression effects [39]. Cytomix significantly increased the level of CD200 on iMSCs WT, but not on the other types of MSCs. CD119, as a subtype of IFN-γ receptor, is a biomarker on BM-MSCs that related to donor gender and MSC suppression of T cell proliferation [35]. CD120b, also named TNF receptor 2 (TNFR2), has also been demonstrated to be an active immune checkpoint for MSC suppression of the activation and proliferation of T cells [40]. Cytomix significantly increased the expression level of CD119 and CD120b on BM-MSCs but not on the two types of iMSCs. On the contrary, Cytomix significantly increased the expression of CD274 (PD-L1), which plays a crucial role in MSC inhibition on T cell activation [41], on iMSCs WT and iMSCs B2M KO, but not on BM-MSCs. In summary, the results indicate that Cytomix preconditioning can promote the immunomodulation of BM-MSCs, iMSCs WT and iMSCs B2M KO on macrophages and T cells by inducing the expression of MHC class I antigens and immunomodulatory markers.

The MSC secretome consists of various soluble factors such as cytokines, chemokines, growth factors, DNA and RNA molecules, all of which play vital roles in MSC immunomodulatory effects in human diseases [42,43]. MSC preconditioning with different inflammatory factors or hypoxia can enhance the therapeutic effects of the MSC secretome [43]. In this study, Cytomix pre-activation was found to change the secretome of BM-MSCs, iMSCs WT and iMSCs B2M KO. Naïve BM-MSCs, iMSCs WT and iMSCs B2M KO produce no IL-8, IL-6 or MCP-1. Cytomix pre-activation significantly increased the secretion of IL-8, IL-6 and MCP-1 from the three types of MSCs analysed. Among the Cytomix-stimulated MSCs, a significantly higher level of IL-6 was detected in the CM from BM-MSCs and a significantly lower level of MCP-1 was detected in the CM from iMSCs B2M KO. A previous study found that IL-8 from MSCs reduced the efficacy of MSCs used to treat liver failure [44]. IL-6 plays an important role in MSC inhibition of lymphocyte apoptosis and in MSC mediation of IL-10 production by macrophages [45,46]. MCP-1 from MSCs was reported to promote T cell proliferation [47]. In contrast, Cytomix exposure significantly decreased the levels of TNFR1 and TGF-β1, which regulate immune response. In the CM from naïve MSCs, iMSCs WT secreted the highest level of TNFR1 and TGF-β1. Moreover, VEGF plays an important role in regulating angiogenesis. Cytomix had a trend to increase the level of VEGF secreted by BM-MSC while significantly decreasing VEGF from iMSCs WT. Interestingly, iMSCs B2M KO did not secrete VEGF with or without Cytomix stimulation. However, the factors measured have complex function in immune response, so different functional assays including an antimicrobial assay, phagocytosis assay and T cell proliferation assay were conducted to test MSC secretome therapeutic potencies.

MSCs have shown direct antimicrobial effects through their secretome [48,49]. The bacterial proliferation results showed that Cytomix enhanced all three types of MSC inhibitory effects on the proliferation of *E. coli*, and enhanced BM-MSCs’ anti-*K. pneumoniae* effects and maintained all of the MSCs’ anti-*S. aureus* effects. The results suggest that soluble factors from MSCs strongly inhibit Gram-positive bacterium growth no matter their sources or activation status, and that proinflammatory cytokines can increase the antimicrobial capacities of both BM-MSCs and iMSCs on certain Gram-negative bacteria. In addition, the antibacterial results indicate that different types of MSCs may exert differential inhibition of different microbes, making the in vitro tests important for MSC applications in infection and inflammation.

Macrophages mediate both innate and adaptive immunity through phagocytosis and the release of inflammatory mediators [3]. We found that the CM from BM-MSCs, iMSCs WT and iMSCs B2M KO significantly inhibited the NF-κB activation in THP-1 macrophage-like cells, while Cytomix reduced the MSCs’ inhibitory effects, which may be related to the inflammatory cytokines in the CM such as IL-6, IL-8 and MCP-1. In addition, Cytomix-stimulated MSCs significantly increased the phagocytosis capacity of differentiated THP-1 cells. These results indicate that the secretome of BM-MSCs, iMSCs WT and iMSCs B2M KO can modulate macrophage inflammatory response, and the proinflammatory cytokines can play a dual role in the MSCs’ influence on macrophages.

There are two types of T cells (CD3+) that are typically investigated: helper T cells (CD4+), which regulate cellular and humoral immune responses, and cytotoxic T cells (CD8+), which kill the cells infected by microbes or cancerous cells [3]. MSCs can suppress T cell activation and proliferation by cell–cell contact, production of soluble mediators and induction of regulatory T cells [50]. In this T cell proliferation model, we studied the expansion index (EI), which is the total number of cells divided by cells at the start of culture, and the division percentage (DP), which represents the amount of T cells that go into cell division. The effects of both the MSC secretome and the cell-to-cell direct contact of MSCs with PBMCs on T cell proliferation were investigated. The results show that the secretome of all of the Cytomix-preconditioned MSCs significantly reduced the expansion of CD3+ and CD3+CD4+ T cells. Cytomix enhanced the iMSCs WT and iMSCs B2M KO inhibition of CD3+ and CD3+CD4+ T cells expansion and iMSCs B2M KO inhibition of CD3+CD8+ T cell expansion. In addition, co-culture of the MSCs with PBMCs significantly decreased DP, and the EI of CD3+, CD3+CD4+ and CD3+CD8+ T cells with a dose-dependent effect, without influence by naïve or proinflammatory MSC precondition. It is well known that MSC immunosuppression is produced through both paracrine signalling and direct cell–cell contact mechanisms, including adhesion molecules and immune checkpoints [51]. The inhibitory effect observed in direct co-culture is stronger than the effect that has been observed in the culture with the MSC secretome. At the ratio of 1:20 (MSC: PBMC), iMSCs WT showed the strongest inhibition of the three types of MSCs, suggesting a potential saturation effect in co-culture conditions [52]. The results showed an increase in G-CSF and a reduction in IFN-γ levels in the co-culture system with MSCs having dose-dependent effects. G-CSF secreted by macrophages can react with G-CSF receptor expressed on T cells, inhibiting T cell proliferation and activation via downregulating the production of INF-γ [53]. MSCs are highly responsive to their microenvironment, and their exposure to activated PBMCs can dynamically prime the MSCs, thereby reducing functional differences between naïve and preconditioned cells [54]. These results suggest that cell–cell contact is the main mechanism of MSC inhibition of T cell proliferation; the potential mechanisms involve the MSC production of G-CSF and the downregulation of IFN-γ.

In summary, while BM-MSCs, iMSCs WT and iMSCs B2M KO share core immunomodulatory functions, they display distinct immunophenotypic and functional profiles. Notably, iMSCs B2M KO exhibit a unique low-immunogenic phenotype due to the absence of HLA-ABC expression, whereas BM-MSCs show higher immunogenicity and proinflammatory cytokine production. In contrast, iMSCs WT demonstrate superior T cell-suppressive capacity. These differences highlight that, despite functional overlap, each MSC type may be better suited for specific therapeutic contexts depending on the desired balance between immunogenicity and immunosuppressive potency. Furthermore, exposure to proinflammatory factors modulated the phenotype and functions of all of the MSC types, influencing their immunogenicity, anti-inflammatory properties, antimicrobial capacity, and immunomodulatory effects on macrophages and T-cells.

There were some limitations in this study. The soluble factors measured do not represent the full profile of the MSC secretome. A wider analysis of the secretome is needed to further investigate the changes in MSC products after preconditioning with Cytomix. Also, the macrophages used in this study were differentiated from a THP-1 cell line which might behave differently than primary tissue resident macrophages or monocyte-derived macrophages. Primary macrophages should ideally be used to investigate MSC immunomodulation on this cell type, depending on the different pathophysiology of inflammation. Moreover, additional in vitro assays regarding MSC modulation on neutrophils, dendritic cells and NK cells are required to provide a more complete picture of the MSC immunomodulatory effects.

## 4. Materials and Methods

### 4.1. Cell Culture

#### 4.1.1. Bone Marrow-Derived MSC (BM-MSC)

Human bone marrow aspiration was obtained from the Clinical Research Facility at University Hospital Galway. BM-MSCs were isolated using an in-house standardised guideline [12,29]. Briefly, 5 × 10^4^ mononuclear cells/cm^2^ were seeded to T175s in complete minimum essential medium alpha (MEM-α, Cat. # 32561029, Thermo Fisher Scientific, Waltham, MA, USA) with 10% foetal bovine serum (FBS, Cat# F7524-500ML; Sigma-Aldrich Ireland Ltd., Arklow, Ireland), 1% penicillin–streptomycin (P/S, Cat# 15140-122; Thermo Fisher Scientific), and 2.5 ng/mL fibroblast growth factor 2 (FGF-2, Cat# 11343625; ImmunoTools, Friesoythe, Germany). When the cells reached 70–80% confluence, they were trypsinised using Trypsin-EDTA (Cat# 25200056; Thermo Fisher Scientific), washed, and cryopreserved in freezing media made with 90% FBS and 10% dimethyl sulfoxide (DMSO, Cat# D2653-5X5ML; Sigma-Aldrich Ireland, Arklow, Ireland). These BM-MSCs were characterised following the International Society for Cell and Gene therapy (ISCT) standard with positive expression of CD105, CD73 and CD90 and negative expression of CD45, CD19/CD79α, CD14/CD11b, CD34, and HLA-DR [29]. BM-MSCs used in experiments were at passage 3, maintained at 37 °C, 95% humidity, 5% CO_2_ and 2% O_2_ and cultured in a serum-free medium (Mesencult complete medium): MesenCult™-ACF Plus Medium (Cat# 05446; STEMCELL Technologies Canada Inc., Vancouver, BC, Canada) plus MesenCult™-ACF 1X Supplement (Cat# 05446, STEMCELL Technologies Canada), GlutaMAX 1X (100X, Cat# 35050-038; Thermo Fisher Scientific) and 1% P/S.

#### 4.1.2. Human Induced Pluripotent Stem Cell-Derived MSCs Wild Type (iMSCs WT)

Human wild type iMSCs (iMSCs WT) were developed and provided by Factor Bioscience (Boston, MA, USA) under a collaborative agreement. Briefly, human dermal fibroblasts used for reprogramming were obtained via punch biopsy. All procedures were conducted in accordance with relevant guidelines and regulations. Adult fibroblasts from a skin biopsy from a healthy donor were isolated and cultured following donor consent. Human dermal fibroblasts were reprogrammed to pluripotency using mRNA encoding Oct4, Sox2, Lin28, cMyc (T58A) and Klf4 complexed with Lipofectamine 3000 (Invitrogen) according to the following transfection schedule: 0.25 µg (D0), 1 µg (D3, D4), and 2 µg (D6-D8). Pluripotent colonies were visible approximately 12 days after the first transfection, and on D15, colonies were manually isolated using a disposable flat-blade cell lifter. iMSCs were generated by using the STEMdiff Mesenchymal Progenitor Kit (Cat# 05240; STEMCELL Technologies Canada) following the manufacturer’s instructions. The cryovial of iMSCs WT was received de-identified at passage 5 and cultured as per the supplier’s protocol in Mesencult complete media. All of the plasticware used was pretreated with Animal Component-Free Cell Attachment Substrate (1:300, Cat# 07130; STEMCELL Technologies Canada) for 2 h and washed with DPBS. Human iMSCs WT used in the following experiments were at passage 9, maintained at 37 °C, 95% humidity, 5% CO_2_ and 2% O_2_ and cultured in Mesencult complete medium.

#### 4.1.3. Beta 2 Microglobulin-Knockout iMSC (iMSC B2M KO)

Beta 2 microglobulin-knockout iMSCs (iMSCs B2M KO), which lack HLA class I molecule expression, were provided and characterised by Factor Bioscience. Briefly, the B2M gene was knocked out in iPSCs by introducing a biallelic 14 bp deletion at exon 2 of the B2M gene using mRNA encoding NoveSlice™ (Factor Bioscience; Cambridge, MA, USA; custom-made, not commercially available), a proprietary dimerizing chromatin-context-sensitive gene-editing endonuclease. Following knockout verification via amplicon sequencing, iPSCs B2M KO were differentiated to iMSCs B2M KO using the previous protocol. The cryovial of iMSCs B2M KO was received de-identified at passage 5 and cultured as per the manufacturer’s instructions in Mesencult complete media on flasks or plates pretreated with the Cell Attachment Substrate. Human iMSCs B2M KO used in experiments were at passage 9, maintained at 37 °C, 95% humidity, 5% CO_2_ and 2% O_2_ and cultured in Mesencult complete medium.

BM-MSCs, iMSCs WT and iMSCs B2M KO were characterized with BD Stemflow™ Human MSC Analysis Kit (BD Biosciences, San Jose, CA, USA) for MSC-positive and MSC-negative markers [12].

#### 4.1.4. THP1-Blue™-CD14 Cell Line

THP1-Blue™-CD14 cells (Cat# thp-cd14sp; InvivoGen, Toulouse, France) are derived from the human acute monocytic leukaemia cell line (THP-1) with stable transfection of a reporter plasmid expressing a secreted embryonic alkaline phosphatase (SEAP) gene under the control of a promoter inducible by the transcription factors NF-κB and AP-1. THP1-Blue™-CD14 cells were used within passage 20 and cultured in Roswell Park Memorial Institute (RPMI) growth medium with L-glutamine (Cat# R8758; Sigma-Aldrich Ireland) supplemented with 10% FBS, 1% P/S and 100 μg/mL normocin™ (Cat#. Ant-nr-1; InvivoGen). Cells were passaged every 3–4 days, and the cell concentration was not allowed to exceed 2 × 10^6^ cells/mL. The cells were selected every two passages by adding 200 μg/mL of Zeocin (Cat# ant-zn-1; InvivoGen) and 250 μg/mL of G418 (Cat# ant-gn-1; InvivoGen) to the growth media. All cells were maintained at 37 °C, 95% humidity, 5% CO_2_ and ambient O_2_.

#### 4.1.5. Peripheral Blood Mononuclear Cells (PBMCs)

Peripheral blood mononuclear cells (PBMCs) were isolated from buffy coat obtained from the Irish Blood Transfusion Service (IBTS. PBMCs were isolated through Histopaque^®^-1077 (Cat# 10771; Sigma-Aldrich Ireland) density gradient as per the manufacturer’s instruction. Briefly, the buffy coat, Histopaque^®^-1077 and DPBS were equilibrated to room temperature (RT). The buffy coat was diluted with DPBS and carefully layered onto Histopaque^®^-1077 without disturbing the liquid. The samples were centrifuged at 400× *g* for 30 min at 20 °C and brakes set to 0. After centrifugation, the opaque interface layer of mononuclear cells was collected, washed with 30 mL DPBS and centrifuged at 250× *g* for 10 min at 20 °C. PBMCs were cryopreserved in freezing media and kept in liquid nitrogen for further usage.

### 4.2. Cytomix Stimulation and Conditioned Media Collection

Cytomix is a cocktail of proinflammatory cytokines including IL-1β, IFN-γ and TNF-α (Cat# 11340015, 11343536, 11343015; ImmunoTools), at 50 ng/mL each, in Mesencult complete media. BM-MSCs, iMSCs WT and iMSCs B2M KO were cultured until 70% confluency, when media was changed to fresh complete media without Cytomix (naïve) or with Cytomix for 24 h. Then, the cells were harvested for flow cytometry or washed twice with DBPS and cultured in Mesencult basal media plus Glutamax for another 24 h. The conditioned media (CM) were collected, aliquoted and stored at −80 °C for further experiments.

### 4.3. MSC Immunophenotype Characterisation

After being exposed to fresh media with or without Cytomix for 24 h, BM-MSCs, iMSCs WT and iMSCs B2M KO were trypsinised and collected for immunophenotype characterisation. The cells were centrifuged at 300× *g* for 5 min and resuspended in 100 µL FACS buffer containing anti-human HLA-ABC-VioGreen, HLA-DR-VioBlue, CD54-APC, CD274-PE-Vio^®^ 615, CD119-FITC (Cat# 130-120-436, 130-111-794, 130-121-342, 130-122-811, 130-099-931; Miltenyi Biotec, Bergisch Gladbach, Germany), CD120b-PE and CD200-PE-CY7 antibodies (Cat# 358403, 399805; BioLegend, San Diego, CA, USA). The cells were incubated with antibodies for 30 min at 4 °C in the dark and washed once with FACS buffer before being incubated with DRAQ7 (Cat# 424001; BioLegend) for 10 min at room temperature in the dark. The antibodies were titrated and used as per manufacturer’s instructions (Supplementary Table S1). Flow cytometry was performed using Northern Lights 3000 (Cytek Biosciences, Fremont, CA, USA). Data were analysed using Flowjo software, version 10.9 (BD Bioscience).

### 4.4. MSC Secretome Profile

The CMs were used to profile the secretome from BM-MSCs, iMSCs WT and iMSCs B2M KO. Proinflammatory cytokines including IL-8, IL-6 and monocyte chemoattractant protein (MCP)-1, and anti-inflammatory/pro-repair cytokines including tumour necrosis factor receptor (TNFR)1, transforming growth factor (TGF)-β1, and vascular endothelial growth factor (VEGF) were measured in their CM using ELISAs (Cat# DY208, DY206, DY279, DY225, DY240, DY293b; R&D Systems, Minneapolis, MN, USA) as per the manufacturer’s instructions.

### 4.5. Bacterial Proliferation Assay

*E. coli* #25932 that was sourced from ATCC (Manassas, VA, USA), *S. aureus* #5624 that was provided by the Discipline of Clinical Microbiology, University Hospital Galway, Ireland, and *K. pneumoniae* #2119 that was provided by Tullamore General Hospital, Ireland were tested for proliferation in the presence of CM as previously described [55]. In brief, bacterial beads were seeded and incubated at 37 °C and 180 RPM for 16 h during the logarithmic growth phase. The bacteria were pelleted and resuspended in DPBS, and optical density was read at 595 nm. Bacterial concentrations were calculated from a previously determined standard curve specifically for each bacterium. An aliquot of 10 µL of each bacterium at 2 × 10^5^ bacteria/µL and 190 µL of CM from naïve or Cytomix-stimulated BM-MSCs, iMSCs WT and iMSCs B2M KO were cultured together for 4 h at 37 °C and 180 RPM. After being spun down and resuspended in 200 µL of DPBS, absorbance at 595 nm was read with SpectraMax^®^ M5 (Molecular Devices, San Jose, CA, USA).

### 4.6. THP-1-Derived Macrophage Quanti-Blue and Phagocytosis Assay

THP1-Blue™-CD14 cells were seeded at 5 × 10^4^ cells/well in 100 µL RPMI growth media containing 20 ng/mL phorbol 12-myristate 13-acetate (PMA) in a 96-well plate and cultured for 48 h. The cells were washed with DPBS once and cultured in a 100 µL mixture of CM and RPMI complete growth media (CM:RPMI = 3:1) containing 100 ng/mL LPS (Cat# tlrl-pb5lps InvivoGen) for 24 h. NF-κB activation was assessed using the THP1-Blue reporter system, in which NF-κB-driven secretion of embryonic alkaline phosphatase (SEAP) is quantified. Following this, 20 µL of the media was transferred to a 96-well plate containing 180 µL of Quanti-Blue working reagents (Cat# tlrl-eklps; InvivoGen). The plate was incubated at 37 °C and absorbance read at 655 nm at 5 h using SpectraMax^®^ M5, with absorbance directly proportional to NF-κB transcriptional activity. The remainder of the media was aspirated and 100 µL RPMI complete media containing 100 ng/mL LPS were added, and the cells were incubated for a further 4 h for the phagocytosis assay.

*S. cerevisiae* Zymosan A BioParticles-FITC (Cat# Z2841; Thermo Fisher Scientific) constituted in DPBS were opsonized with human serum at 2 mg/mL and incubated for 1 h at 37 °C with regular agitation. The particles were washed twice with DPBS and resuspended in RPMI complete media before being added to the cells at 8 particles per cell. The cells were incubated at 37 °C for 30 min, then washed twice with DPBS before being fixed in 4% PFA for 10–15 min at RT. After fixation, the cells were washed twice and kept in DPBS, and fluorescence images were acquired using the Cytation 1 Cell Imaging Multi-Mode Reader (BioTek Instruments, Winooski, VT, USA). A macrophage with more than 2 intracellular particles was considered positive for phagocytosis. The phagocytosis-positive percentage of total cells was calculated by counting more than 300 cells in total across 3 images.

### 4.7. T Cell Proliferation Assay

Cryopreserved PBMCs were thawed, washed once with DPBS, counted and resuspended in DPBS at a final concentration of 2 × 10^7^ cells/mL. The cells were stained with 1 µM CellTrace™ CFSE (Cat# C34554, Thermo Fisher Scientificfor 5 min at RT, protected from light. RPMI-1640 complete medium was added to saturate the remaining CFSE with 5 min incubation at RT.

To test the effects of the MSC secretome on T cell proliferation, the stained PBMCs were centrifuged at 300× *g* for 5 min and resuspended in a 1:1 mixture of CM and RPMI-1640 complete medium containing phytohemagglutinin (PHA, Cat# L8754; Sigma-Aldrich Irelandat 10 µg/mL and IL-2 (Cat# 11340023; ImmunoTools) at 100 IU/mL.

To test the direct effects of the MSCs on T cell proliferation, three different MSC cell densities were plated (2 × 10^3^, 5 × 10^3^ and 1 × 10^4^ MSCs/well) on a flat-bottomed 96-well plate and pre-stimulated with PBS or Cytomix for 24 h. Then, media was removed, cells were washed once with DPBS and 1 × 10^5^ of stained PBMCs were added per well in 100 µL RPMI complete media containing 10 µg/mL of PHA and 100 IU/mL of IL-2 to MSCs.

After 96 h of culture, the PBMCs were pelleted at 300× *g* for 5 min, and the supernatant was collected to measure TNF-α, IFN-γ, IL-10 and granulocyte colony-stimulating factor (G-CSF) with ELISA kits (Cat# DY210, DY285B, DY217B; R&D Systems). The PBMCs were stained with anti-human CD3-APC-Vio770, CD4-APC and CD8-PE-Vio770 antibodies (Cat# 130-113-136, 130-113-222, 130-110-680; Miltenyi Biotec) for 30 min on ice in the dark. After being washed with FACS buffer, the cells were incubated with 7-AAD (Cat# 130-111-568; Miltenyi Biotec) for 5 min at room temperature. Quantitative flow cytometry was performed using Northern Lights 3000. Data were analysed using Flowjo software, version 10.9.

### 4.8. Statistical Analysis and Data Processing

Statistical analysis was performed using GraphPad Prism, version 9.0.1 (GraphPad Software, San Diego, CA, USA). The results were presented as the mean ± standard deviation (SD). All data were analysed for normality using the Shapiro–Wilk test. After the normality of data was confirmed, the results were grouped by two experimental factors: priming (naïve vs. Cytomix) and cell type (BM-MSCs vs. iMSCs WT vs. iMSCs B2M KO). Two-way ANOVA analysis followed by the Šídák’s multiple comparisons test (to compare naïve vs. Cytomix in each type of MSCs) or Tukey’s multiple comparisons test (to compare BM-MSCs vs. iMSCs WT vs. iMSCs B2M KO in the naïve or Cytomix group) or Dunnett’s multiple comparisons test (to compare BM-MSCs or iMSCs WT or iMSCs B2M KO vs. vehicle in the naïve or Cytomix group) was performed; a *p* value < 0.05 was considered statistically significant.

## Data Availability

The datasets generated and/or analysed during the current study are not publicly available but are available from the corresponding author on reasonable request.

## Supplementary Materials

The following supporting information can be downloaded at: https://www.mdpi.com/article/10.3390/ijms27094090/s1.

## Abbreviations

The following abbreviations are used in this manuscript:

MSC	Mesenchymal stromal cell
ARDS	Acute respiratory distress syndrome
BM-MSC	Bone marrow-derived MSC
iMSC WT	Human induced pluripotent stem cell-derived MSC wild type
iMSC B2M KO	β2 microglobulin-knockout iMSC
IBD	Inflammatory bowel diseases
GvHD	Graft-versus-host disease
ISCT	International Society for Cellular Therapy
HLA	Human leukocyte antigen class I complex
IL	Interleukin
IFN	Interferon
TNF	Tumour necrosis factor
UC-MSC	Umbilical cord-derived MSC
MFI	Median fluorescence intensity
EI	Expansion index
DP	Division percentage
MEM	Complete minimum essential medium
DMSO	Dimethyl sulfoxide
RPMI	Roswell Park Memorial Institute
PBMC	Peripheral blood mononuclear cell
IBTS	Irish Blood Transfusion Service
CM	Conditioned media
MCP	Monocyte chemoattractant protein
TNFR	Tumour necrosis factor receptor
TGF	Transforming growth factor
VEGF	Vascular endothelial growth factor
PMA	Phorbol 12-myristate 13-acetate
G-CSF	Granulocyte colony-stimulating factor
SD	Standard deviation
