# Supplementary material for: Proinflammatory Cytokine Preconditioning Enhances the Therapeutic Potency of Different Types of MSCs in Inflammation
Source: Int J Mol Sci. 2026 May 2;27(9):4090. doi: 10.3390/ijms27094090 (PMC13164479; doi:10.3390/ijms27094090)
Supplement: Supplementary file 1 [file ijms-27-04090-s001.zip › ijms-4277664-supplementary.pdf]

Supplementary Table S1: Antibodies used in flow

Antibody	Company	Cat no	Dilution
HLA-ABC-VioGreen (human)	Miltenyi	130-120-436	1:50
HLA-DR-VioBlue (human)	Miltenyi	130-111-794	1:50
CD54-APC (human)	Miltenyi	130-121-342	1:50
CD200-PE-CY7 (human)	BioLegend	399805	1:20
CD119-FITC (human)	Miltenyi	130-099-931	1:33
CD120b-PE (human)	BioLegend	358403	1:20
CD274-PE-Vio® 615 (human)	Miltenyi	130-122-811	1:167
DRAQ7	BioLegend	424001	1:900
CD3-APC-Vio770 (human)	Miltenyi	130-113-136	1:100
CD4-APC (human)	Miltenyi	130-113-222	1:50
CD8-PE-Vio770 (human)	Miltenyi	130-110-680	1:50
7-AAD	Miltenyi	130-111-568	1:50

Supplementary figure S1

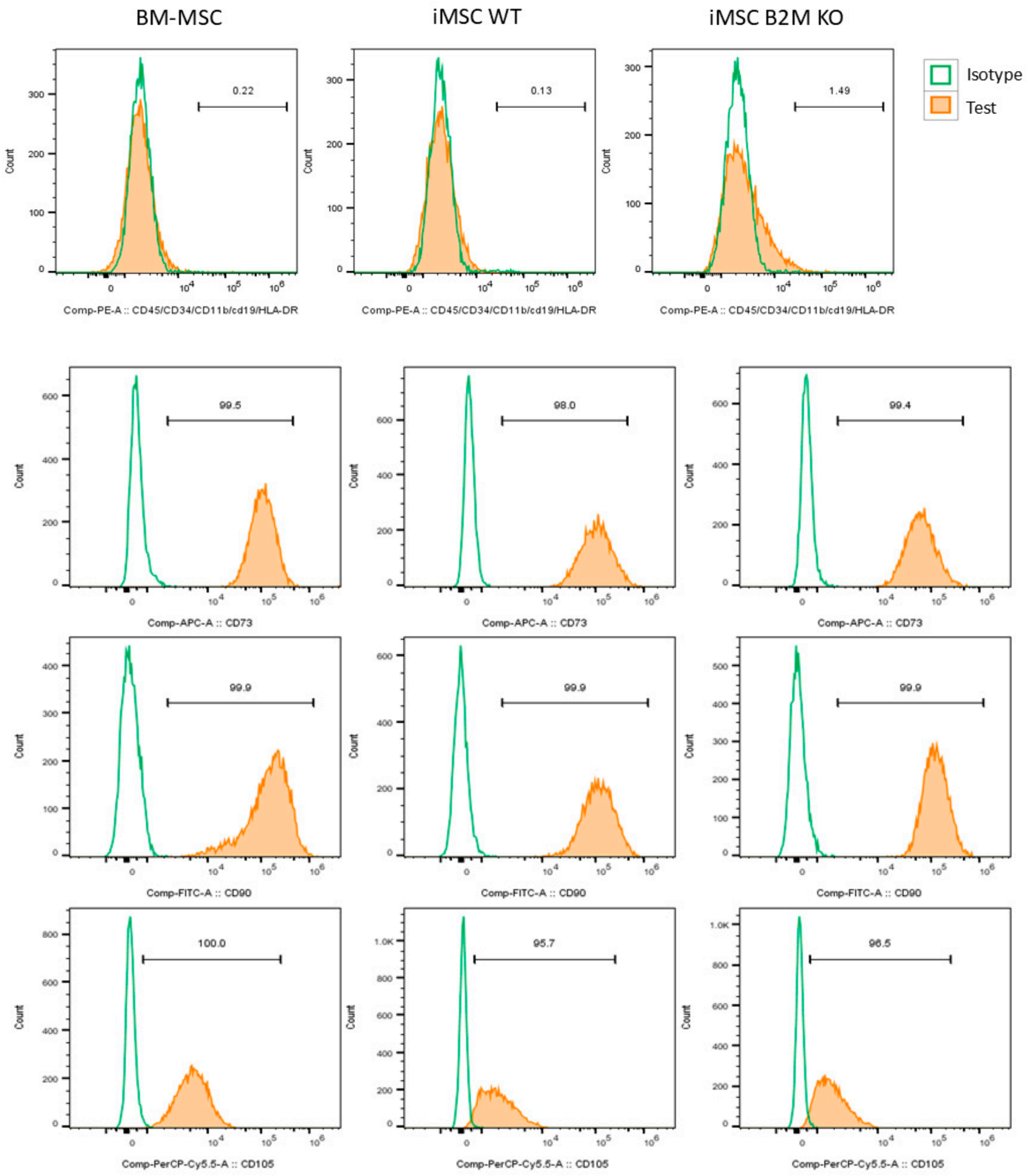

MSC marker characterisation: BM-MSCs, iMSC WT, and iMSC B2M KO were ⩾95% positive for CD73, CD90, and CD105, and ⩽2% positive for CD45, CD34, CD11b, CD19, and HLA-DR.

Supplementary figure S2

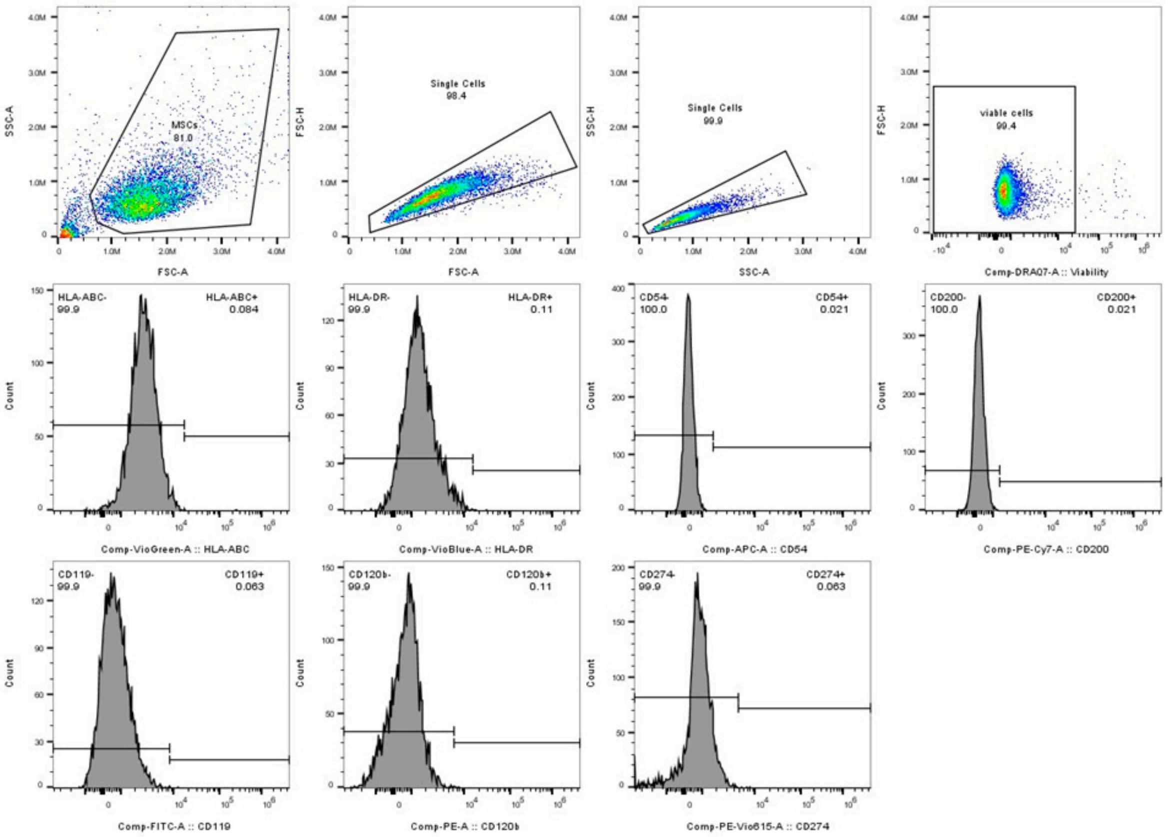

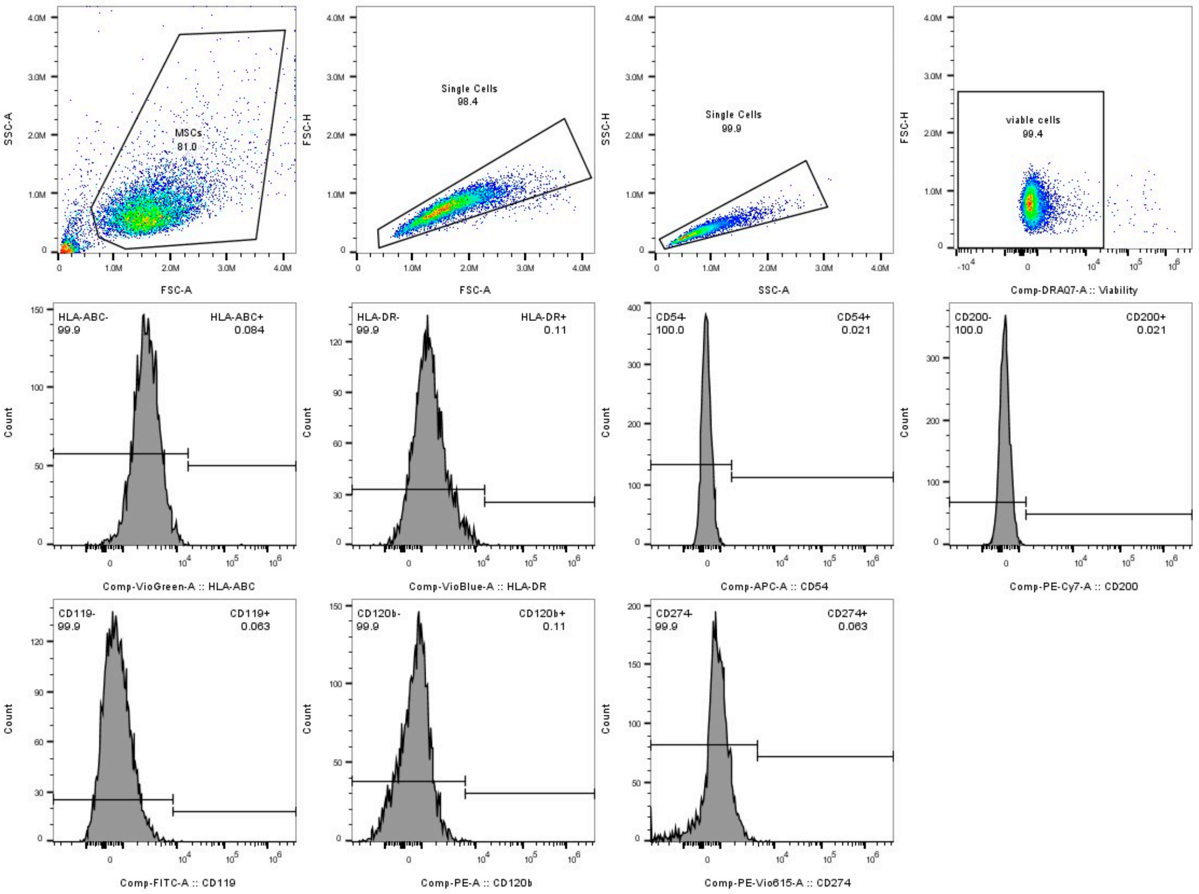

Representation of the gating strategy for MSC immunophenotyping using fluorescence minus one (FMO) controls. Flow cytometry data were analysed using FlowJo software, version 10.9.

Supplementary figure S3

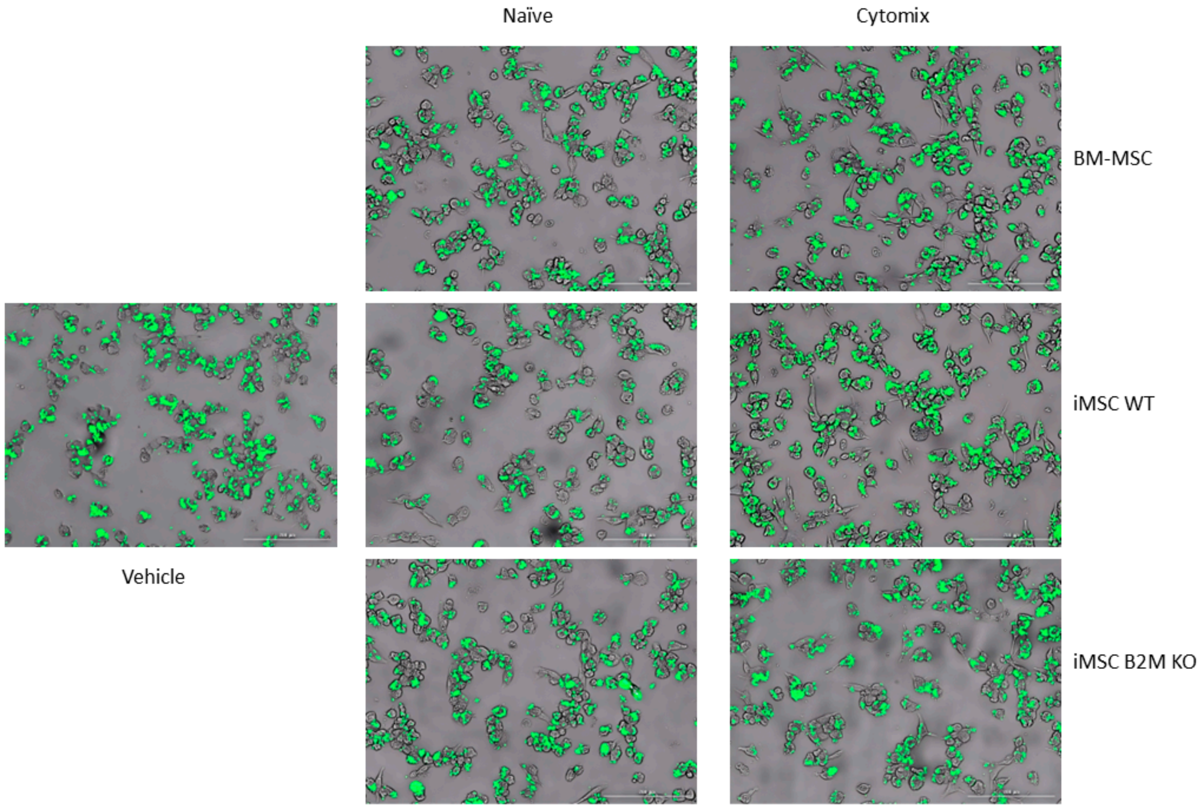

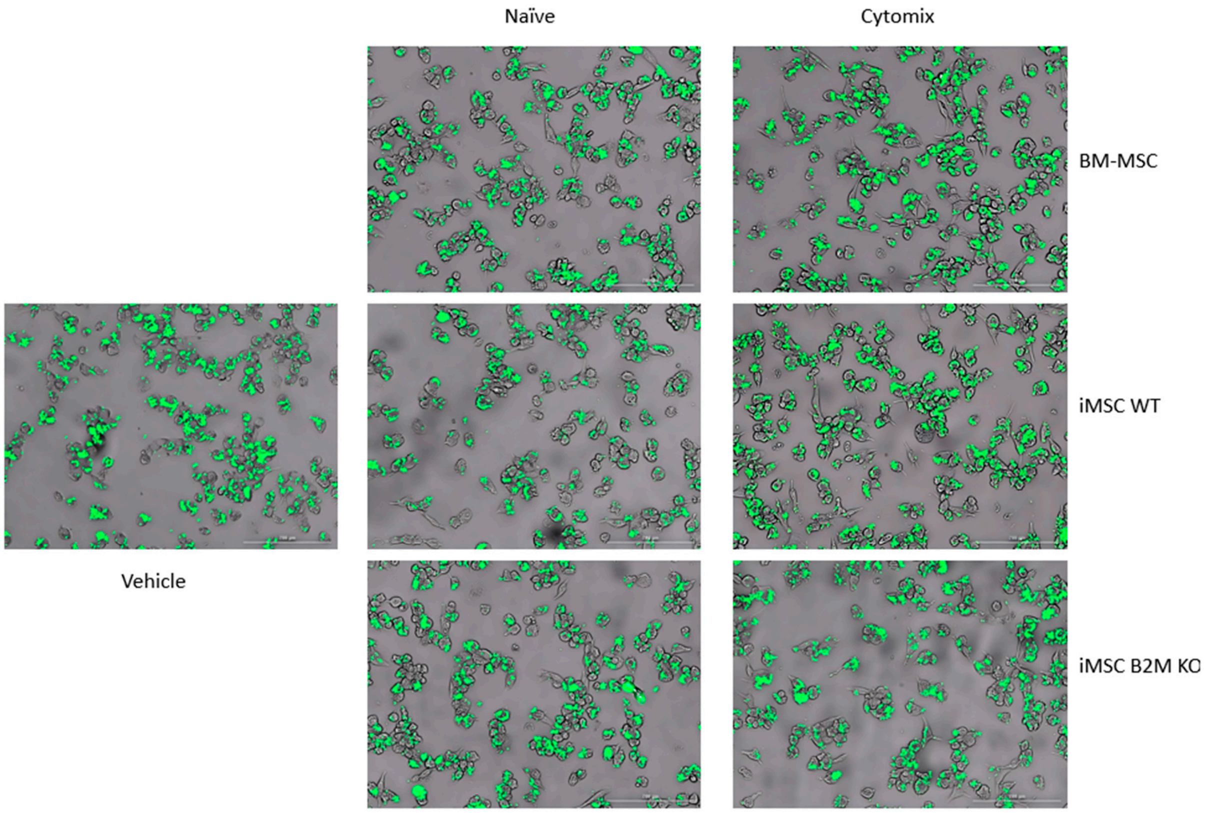

Representative phagocytosis photomicrographs for each group. Macrophages containing more than two intracellular particles were considered positive. White bar in photomicrographs indicates a scale of 200 μm.

Supplementary figure S4

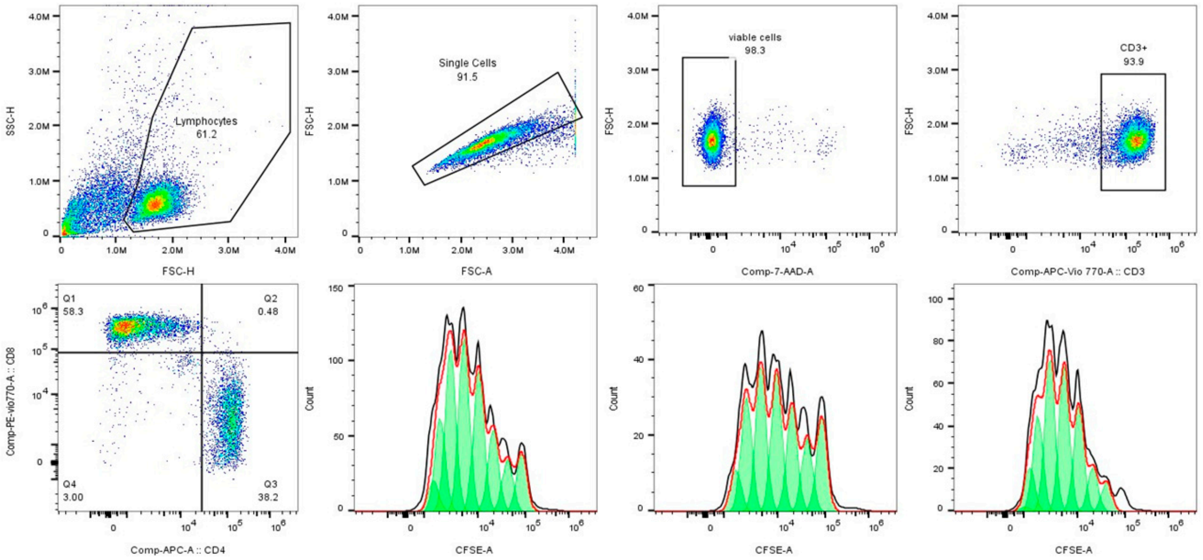

Gating strategy for T cell proliferation. Flow cytometry data were analysed using FlowJo software, version 10.9. Division percentage and expansion index were calculated using the proliferation model.
